# The impact of the COVID-19 pandemic on musculoskeletal disorders-related sick leave among healthcare workers: a retrospective analysis of Slovenian national data

**DOI:** 10.3389/fpubh.2024.1478204

**Published:** 2025-01-06

**Authors:** Dorjana Zerbo Šporin, Žiga Kozinc, Ticijana Prijon, Tanja Metličar, Nejc Šarabon

**Affiliations:** ^1^Faculty of Health Sciences, University of Primorska, Izola, Slovenia; ^2^University of Primorska, Andrej Marušič Institute, Koper, Slovenia; ^3^National Institute of Public Health, Ljubljana, Slovenia; ^4^Community Health Center Ljubljana, Ljubljana, Slovenia; ^5^Ludwig Boltzmann Institute for Rehabilitation Research, Saint Pölten, Austria

**Keywords:** absenteeism, retrospective data, healthcare occupations, healthcare sector, presentism

## Abstract

**Purpose:**

Musculoskeletal disorders (MSDs) are one of the main causes of health-related absenteeism. MSDs were a known problem among healthcare workers (HCWs) even before COVID-19. The pandemic, with its associated stresses and changes in working conditions, may have influenced the incidence and duration of MSDs-related sick leave (SL) among HCWs. The aim of this study was to compare the incidence and duration of MSDs-related SL among HCWs before and during the COVID-19 pandemic, with a focus on differences between age and gender groups.

**Methods:**

A retrospective analysis was conducted using Slovenian national SL data on work-related MSDs within NACE Rev. 2 “Human health activities” for 2019, 2020, and 2021, categorized by gender and age.

**Results:**

The study found that older HCWs, particularly women, consistently had a higher incidence of work-related MSDs SL than their younger counterparts. However, during the pandemic, MSDs were more common among younger men, while the average duration of SL was longer among younger women. On the other hand, in older HCWs, the average SL incidence decreased during the pandemic, while the SL duration substantially increased.

**Conclusion:**

The dynamics of MSDs related SL among HCWs are complex and influenced by several factors, including the challenges posed by the COVID-19 pandemic. Healthcare managers should implement tailored strategies to address MSDs-related absenteeism among specific groups of HCWs to promote a healthier workforce and ensure a resilient healthcare system during health crises.

## 1 Introduction

Musculoskeletal disorders (MSDs) are the main cause of health-related absenteeism in the European Union (42). These illnesses are predominantly caused by physically demanding activities, especially if they are associated with psychosocial challenges (42). In the health and social care sector (HCWs), the prevalence of MSDs is above average: almost a quarter of employees report significant symptoms of MSDs (42, 43, 49). Some studies report an even higher prevalence of MSDs of over 80% among physiotherapists, nurses, midwives, dentists and surgeons (1). The lower back, neck, shoulder and hand/wrist are the body parts most at risk. For surgeons and dentists, the prevalence of MSDs is highest in the lower back (>60%), shoulder and upper limb (35–55%), and for nurses it is >25% for the lower limb (1). Self-reported risk factors for MSDs were: repetitive use of the same posture (29.8%), treating too many patients per day (29.1%), inadequate injury prevention training (15.9%), unfavorable working conditions (11.6%) (2). The healthcare sector reports a higher incidence of psychosocial risks compared to other sectors. Prioritizing the management of MSDs in the workplace was already crucial before the COVID-19 pandemic to ensure sustainable work ability in the healthcare sector (3).

The COVID-19 pandemic, instigated by the SARS-CoV-2 virus, originated in China before spreading rapidly around the world. It was declared a pandemic by the World Health Organization on March 11, 2020 (4). This crisis placed a significant burden on HCWs, exposing them to a higher risk of contracting the virus (44) as well as increased physical, psychological and social challenges (45). Administration of the SARS-CoV-2 vaccine to HCWs showed very high efficacy against infection in the first months after full vaccination, but the efficacy of the vaccine decreases significantly after the emergence of new variants (5). The serological response to the vaccine was rather inadequate in multimorbid HCWs (6). The escalating patient count, coupled with long hours, limited rest, and heightened work stress, amplified the risk of MSDs among HCWs (7). Those with a history of COVID-19 were even more susceptible, especially to low back disorders (7). Studies on HCWs during the COVID-19 pandemic have revealed significant prevalence rates of MSDs. According to cross-sectional research, 54.2–94.9% of HCWs experienced MSDs during the pandemic (8–11), with frontline workers particularly affected, especially in the neck (73.4%) and upper back (61.4%) regions (10). While it is evident that the pandemic exacerbated MSDs in HCWs (12), comprehensive data comparing pre-pandemic and pandemic prevalence and severity remain sparse. Surveys indicate that 22% of 80 cardio-sonographers experienced exacerbated MSDs symptoms during the pandemic (13). Likewise, altered work routines, spurred by pandemic restrictions, intensified the incidence and intensity of the MSDs among 148 UK podiatrists (14).

This retrospective study aimed to contrast the incidence and duration of sick leaves (SL) due to MSDs, pre-and post-COVID-19 outbreak. We sought to discern the pandemic’s impact on MSDs-related SL, with a focus on specific HCW subgroups, based on age and gender. Our hypothesis was that a heightened incidence and prolonged SL due to the pandemic will be observed, especially among older HCWs. Grasping the SL trends in HCWs during health crises, like the COVID-19 pandemic, is vital since MSDs-related absences have been prevalent in this profession before the pandemic. The insights from this study will guide decision-makers in preemptively addressing MSDs risks in potential future pandemics, ensuring a resilient healthcare system.

## 2 Materials and methods

### 2.1 Study population and data collection

We retrospectively analyzed Slovenian national data on SL rates due to the most common work-related MSDs (Table 1) in the “human health activities” division of the NACE Rev. 2 classification of economic activities. The analysis was conducted for the years 2019, 2020, 2021 by gender and age groups: 20.0–44.9 years (younger HCWs), 45.0–64.9 years (older HCWs). Table 2 shows the characteristics of the analyzed sample (i.e., the number of workers in each calendar year for both genders and age groups). The analysis was conducted for 41,292 HCWs in 2019, 45,980 in 2020 and 47,128 in 2021. The data for this study was collected by the Slovenian Institute of Public Health Slovene: Nacionalni inštitut za javno zdravje (NIJZ). The NIJZ collects, analyses and disseminates data on the SL of employees and self-employed persons who are insured under the compulsory health insurance scheme in Slovenia. The source of the data is the certificate of justified absence from work for health reasons (eBOL) and is obtained from healthcare providers. The data collection covers the work force in all economic sectors of NACE Rev. 2 and has a legal basis in the Health Care Databases Act (ZZPPZ - Ur. l. RS 65/00, database NIJZ3) and in the Act on Personal Data Protection (ZVOP-1 - Ur. l. RS 94/07). Article 17 of the Personal Data Protection Act considers scientific research, historical or statistical purposes as lawful processing operations and therefore provides a legal basis for further processing. The sharing of data in this study was also approved by the Ethics Committee of the National Institute of Public Health [approval number: 6310–1/2021–35 (241)]. All data were anonymised at all stages of the study. The study does not contain any data that could be linked to an individual.

**Table 1:** List of the most common work-related MSDs included in the study, by body region.

Body region	Included musculoskeletal disorders
Upper back	Cervical disk disorders (M50.0-M50.9), cervicocranial syndrome (M53.0), cervicobrachial syndrome (M53.1), cervicalgia (M54.2).
Lower back	Other intervertebral disk disorders (M51.0 – M51.9), sciatica (M54.3), lumbago with sciatica (M54.4), low back pain (M54.5).
Shoulder	Adhesive capsulitis of shoulder (M75.0), rotator cuff tear or rupture, not specified as traumatic (M75.1), bicipital tendinitis (M75.2), calcific tendinitis of shoulder (M75.3), impingement syndrome of shoulder (M75.4), bursitis of shoulder (M75.5), other shoulder lesions (M75.8), shoulder lesion, unspecified (M75.9).
Elbow	Medial epicondylitis (M77.0); lateral epicondylitis (M77.1); olecranon bursitis (M70.2); other bursitis of elbow (M70.3).
Hand and wrist	Osteoarthritis of first carpometacarpal joint (M18.0–M18.9); radial styloid tenosynovitis (de Quervain) (M65.4); crepitant synovitis of hand and wrist (M70.0); peri arthritis of wrist (M77.2); carpal tunnel syndrome (G56.0).
Hip	Osteoarthritis of hip (M16.0–M16.9); other articular cartilage disorders of hip (M24.15).
Knee	Osteoarthritis of knee (M17.0–M17.9); internal derangement of knee (M23.0–23.9); prepatellar bursitis (M70.4); other bursitis of knee (M70.5); synovial cyst of popliteal space [Baker] (M71.2).
Ankle	Primary osteoarthritis of ankle and food (M19.07); secondary osteoarthritis of ankle and (M19.27); other and unspecified osteoarthritis (M19).

**Table 2:** Employed persons in in “human health activities” from NACE Rev. 2 classification by age and sex.

	20–44 years			45–65 years			Total
	Men	Women	Total	Men	Women	Total	
2019	6,065	19,780	25,845	3,435	15,447	15,447	41,292
2020	6,263	20,475	26,738	3,511	15,731	19,242	45,980
2021	6,407	21,106	27,513	3,587	16,028	19,615	47,128

### 2.2 Classification of economic activities

According to the NACE Rev. 2 - Statistical Classification of Economic Activities in the European Community, economic activities are divided into 21 sectors. The NACE Rev. 2 Sector Q, “health and social work activities” contains three divisions: “human health activities” (No. 86), “residential care activities” (No. 87) and “social work activities without accommodation” (No. 88). The “human health activities” division analyzed in our study includes short-or long-term hospitals, general or specialized medical activities (15).

### 2.3 Data analysis and outcome measures

For the analysis, we received anonymous data in the form of numbers representing the SL rates for the most common work-related MDSs in “human health activities” by sex and age. The average values of SL rates for 2019, 2020, 2021 were used for the analysis. The role of long-COVID-19 syndrome, SARS-CoV-2 vaccination and previous SARS-CoV-2 infection were not considered. First, we analyzed (i) the frequency of spells (SL incidence), expressed as the number of SL cases (case: one SL in a calendar year from January 1 to December 31, regardless of when SL started) per 100 employees in a year and (ii) the severity of MSDs, expressed as the average duration of one sick leave (SL duration). The number of SL cases is considered as the number of completed SL cases of MSDs in a calendar year (January 1–December 31), regardless of when the SL began. The diseases of the musculoskeletal system or connective tissue according to the International Classification of Diseases ICD-10-AM were considered: dorsopathies (M50-M54), shoulder lesions (M75), soft tissue disorders due to use, overuse and pressure (M70), other enthesopathies (M77), synovitis and tendosynovitis (M65), arthropathies (M00-M25) and carpal tunnel syndrome (G56.0). The most common work-related MSDs by body region included in the study and their classification codes are listed in Table 1. Risk ratios and their 95% confidence intervals were calculated using R (version 4.3.1) with the epi.2by2 function in the epiR package.

## 3 Results

### 3.1 Workers in “human health activities”

The number of employees in the “human health activities” division from NACE Rev. 2 Sector Q, has increased from 41,292 in 2019 to 47,128 in 2021 (Table 2). They account for 5.2% of the Slovenian workforce. More women (79%) than men (21%) are employed in this division. There are about 1.4 times more younger than older HCWs (16).

### 3.2 Sick leave incidence

**Figure 1:**
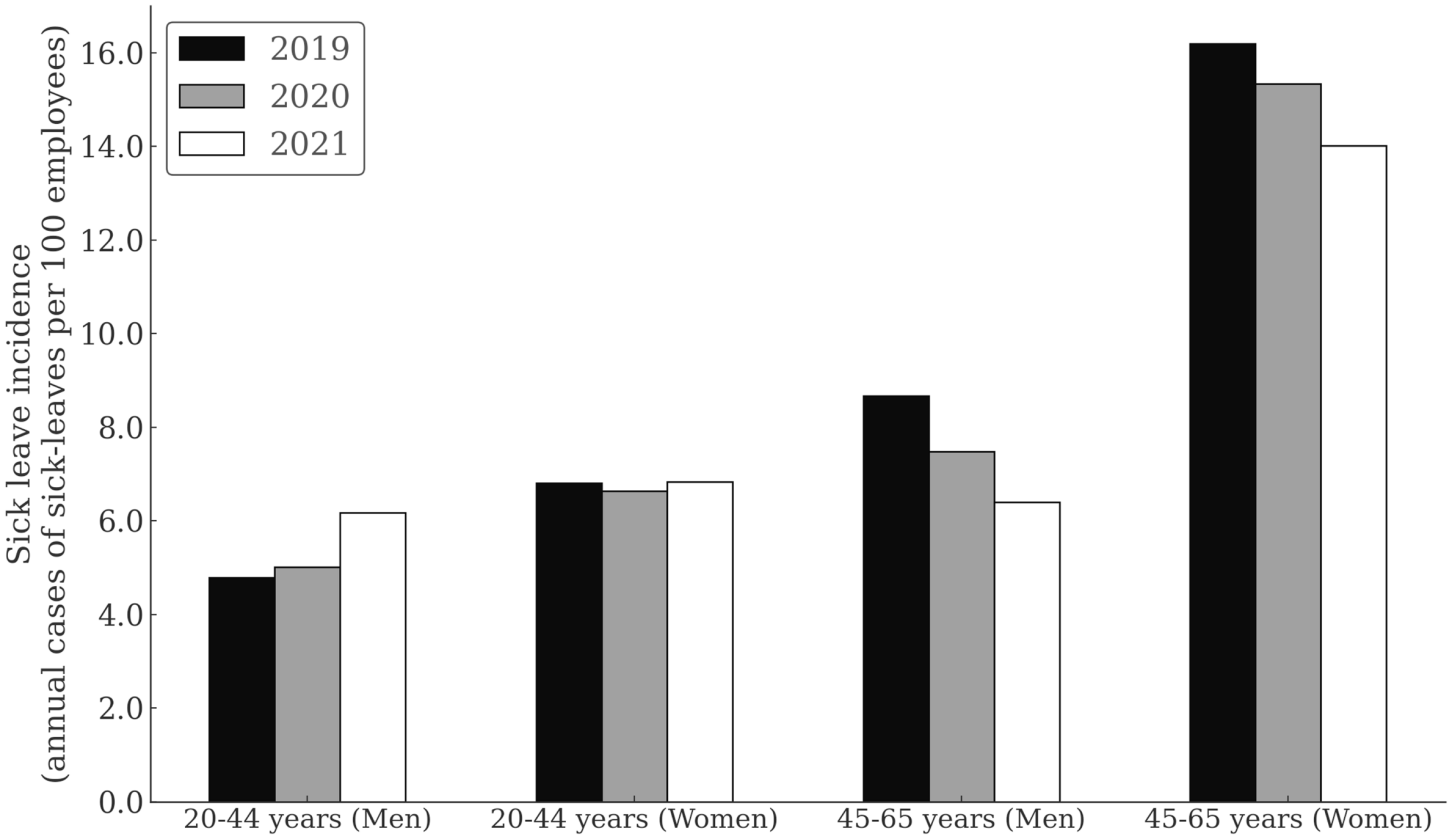
Sick leave incidence duo to musculoskeletal disorders among healthcare workers by sex, age, and calendar year.

Figure 1 and Table 3 show the SL incidence due to MSDs, by sex, age, and calendar year. In general, the incidence of SL was lower in younger compared to the older group, and this was especially pronounced in women.

**Table 3:** Incidence rates (cases per 100 employees) due to MSDs, by sex, age, and calendar year.

Age	Gender	2019	2020	2021
20–44 years	Men	4.8	5.0	6.2
	Women	6.8	6.6	6.8
45–65 years	Men	8.7	7.5	6.4
	Women	16.2	15.3	14.0

Men in younger group had the lowest incidence of SL overall, with 4.79 cases/100 persons in 2019. Compared to 2019, the risk of SL was increased for 29% in 2021 in young men (RR = 1.22; CI = 1.12–1.50; *p* < 0.001). For women in the younger group, the SL incidence was consistent through years (range = 6.64 to 6.83 cases/100 persons). Within the younger group, women had higher SL risk than men in 2019 (RR = 1.42; CI = 1.23–1.85; *p* < 0.001), 2020 (RR = 1.32; CI = 1.18–1.49; *p* < 0.001), but not in 2021 (RR = 1.10; CI = 0.98–1.23; *p* = 0.068).

In the older subgroup, the SL incidence seemed to decline with years. Comparing 2019 to 2021, the risk was higher in 2019, both for men (RR = 1.31; CI = 1.11–1.55; *p* = 0.002) and women (RR = 1.16; CI = 1.10–1.22; *p* < 0.001). In the older age group, the differences in incidence between men and women were even more pronounced, with women exhibiting higher SL incidence compared to men in 2019 (RR = 1.87; CI = 1.67–2.09; *p* < 0.001), 2020 (RR = 2.05; CI = 1.82–2.32; *p* < 0.001) and 2021 (RR = 2.19; CI = 1.92–2.50; *p* < 0.001).

In men, the older subgroup had higher SL incidence than the younger subgroup in 2019 (RR = 1.81; CI = 1.55–2.11; *p* < 0.001), 2020 (RR = 1.49; CI = 1.27–1.74; *p* < 0.001), but not in 2021 (RR = 1.03; CI = 0.88–1.21; *p* = 0.675). In women, older subgroup had higher SL incidence than the younger subgroup in 2019 (RR = 2.38; CI = 2.23–2.53; *p* < 0.001), 2020 (RR = 2.31; CI = 2.17–2.46; *p* < 0.001) and 2021 (RR = 2.05; CI = 1.93–2.18; *p* < 0.001).

### 3.3 Sick leave duration

**Figure 2:**
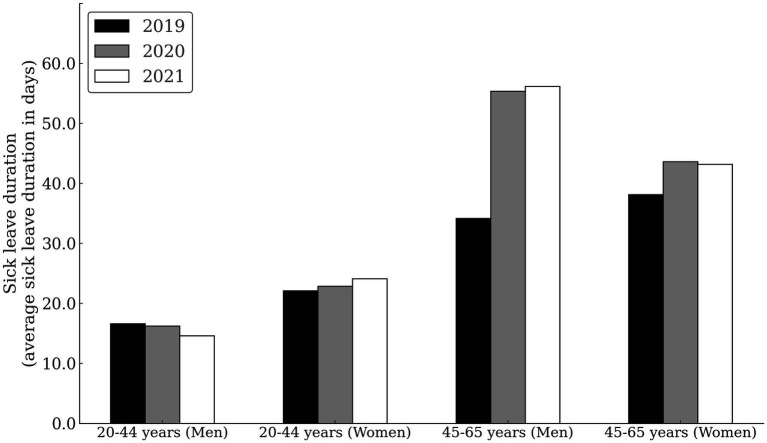
Sick leave duration due to musculoskeletal disorders among healthcare workers by sex, age, and calendar year.

Figure 2 and Table 4 show the average SL duration by sex, age, and calendar year. Younger subgroups had relatively consistent SL durations through years, with a slight decreasing trend in men (16.6 days in 2019, 16.2 days in 2020 and 14.6 days in 2021), and a slight increasing trend in women (22.1 days in 2019, 22.9 days in 2020 and 24.1 days in 2021). Already in 2019, the average SL duration was substantially longer in the older subgroup (34.1 days in men and 38.2 days in women). The average SL duration was further increased in 2020 (55.4 days in men and 43.6 days in women) and 2021 (56.2 days in men and 43.2 days in women).

**Table 4:** Sick-leave severity (average sick-leave duration in days), by sex, age, and calendar year.

Age	Gender	2019	2020	2021
20–44 years	Men	16.6	16.2	14.6
	Women	22.1	22.9	24.1
45–65 years	Men	34.2	55.4	56.2
	Women	38.2	43.6	43.2

## 4 Discussion

We found that regardless of the COVID-19 pandemic, healthcare facilities can expect the highest incidence and duration of MSDs-related SL in older HCWs of both sexes and then in younger women. MSDs are consistently more common in women, and this sex disparity increases with age. However, during the pandemic, an increase in SL incidence is expected in younger men, while young women will have longer sick leave. The SL incidence among older HCWs is lower during the pandemic, but its duration is much longer. Older HCWs are more vulnerable to changes in SL course during the pandemic.

Younger male HCWs had a significantly lower risk of MSDs than women of the same age in 2019 and 2020, but this was not the case in the 2021 pandemic year. At that time, SL incidence was the same in men and women, reflecting an increase in MSDs in younger men. While younger men and women had a similar MSDs related SL incidence, women stayed at SL much longer on average (24.1 days) than men (14.4 days) (Figure 2). From these observations, we conclude that during the pandemic period, an increased incidence of MSDs should be expected in younger male HCWs and a longer SL duration in younger female HCWs. Among younger males, it is interesting to note; that their MSDs-related SL is on average 2 days shorter in 2021 compared with previous years (Figure 2). It is possible that they returned to work during COVID-19 pandemic before they had fully recovered from MSDs and that they are affected by presentiseem. Literature indicates that 13.6% of frontline physicians reported presenteeism (17), which was also found among nurses and respiratory physical therapists (18). Clearly, HCWs feel a duty to patients and their colleagues to work whenever possible (19). Therefore, it is important to control presenteeism in HCWs because its affects individual’s performance and health (20, 21). In MSDs, delaying treatment have a negative impact on health (22), as it increases disease recurrence (23) and treatment costs (24). Therefore, healthcare managers should pay attention to improve the detection and management of presenteeism in emergencies such as pandemic, especially in younger male HCWs.

Similar to other studies (25, 26), we observed that women are more frequently affected by MSDs, with this gender disparity amplifying with age (27, 28) (Figure 1). Women seem to be at an elevated risk for MSDs compared to men (26, 29), even when undertaking similar work tasks (30), and particularly during prolonged repetitive work (29). Given these findings, it’s imperative for managers to prioritize the well-being of female HCWs, especially those in the older age subgroup, to minimize the risk of MSD-related sick leaves, irrespective of any future pandemic scenarios.

Interestingly, the incidence of MSDs sick leave in older HCWs decreases from pre-pandemic to pandemic (Figure 1), whereas the severity of MDSs increases (Figure 2). The decline in SL incidence intensified with each year of the epidemic, with the greatest decline in 2021 compared with 2019 (31% in men and 16% in women) (Figure 1). It is doubtful that the decline in sick leave reflects the decline in MSDs during the COVID-19 pandemic. It is more likely that measures taken during the pandemic to protect people’s health changed the pattern of sick leave. It is also possible that administration workers (recorded as employees in healthcare activities) who worked from home during the pandemics experienced the MSDs symptoms without taking sick leave. Home-based workers were found to have a higher risk of experiencing MSDs than employees in the locations during the first year of COVID-19 pandemic (31). In parallel with a decrease in SL incidence among older HCWs, we can observe an increase in the duration of average SL during the pandemic (Figure 2). On March 13, 2020, the Ministry of Health of the Republic of Slovenia issued the Order on Temporary Measures to Control the Spread of COVID-19 Infectious Disease (Official Gazette of the Republic of Slovenia, No. 22/20 and 32/20), which states that all specialist examinations and surgical procedures will be canceled for all healthcare providers, except for medical services marked as urgent and very urgent, oncology services, and treatment of pregnant women. This measure also applies to rehabilitation and other non-urgent forms of treatment (32). This order expired on 6/1/2020, but the epidemic was proclaimed again on 11/16/2020 and continued into 2021. It is possible that the disruption of access to healthcare services influenced the more difficult course of MSDs, which was particularly evident among older HCWs. The literature reports that confinement had negatively impacted the musculoskeletal health of patients with MSDs. Up to 35% of them reported worsening health-related quality of life after the COVID-19 pandemic (33). Interventions related to preventing the spread of the SARS-CoV-2 virus negatively impacted early treatment and treat-to-target strategies (34) and reduced the quality of life of people with inflammatory rheumatic diseases and MSDs (35). At the beginning of the epidemic, the Slovenian Association of Occupational, Transport and Sports Medicine Association issued a risk assessment and measures on COVID-19, which states, “If it is necessary to designate some workers to wait at home to ensure safe working conditions in the company, we advise giving priority to the more vulnerable groups of workers,” which include older workers and those with more severe diseases [38]. It is possible that these measures have also contributed to older HCWs being less likely to take sick leave for MSDs, as remaining at home could reduce the need for sick leave. Some other aspects of the MSDs pathogenesis in HCWs with COVID-19 need to be emphasized. Studies on the molecular background of acute and chronic pain have linked TRP channels which are responsible for nociception to COVID-19. The pathophysiology in COVID-19 patients is similar to the effects generated by TRPV-1 stimulation. Therefore, TRPV-1 desensitization could be beneficial for the treatment of COVID-19 and its symptoms, as well as back pain that often accompanies COVID-19 infection (36). The incidence of SARS-CoV-2 infection in HCWs varied widely during the different phases of the pandemic. About a quarter of positive HCWs had an asymptomatic infection, especially in those who were partially or fully vaccinated and in subjects with previous infections (37). The long-term consequences of the pandemic remain a major public health priority. In HCWs, different tissues show different rates of aging after SARS-CoV-2 infection, with lung tissue being more susceptible to accelerated aging (38).

The literature comparing sickness absence among HCWs before and during the COVID-19 pandemic is limited and inconsistent. Edge et al. reported a substantial decrease (29.3%) in sickness absence due to MSDs during the first wave of COVID-19 among HCWs in England. A much smaller decrease (4%) from 2019 to 2020 was observed in primary HCWs from Brazil (46). The higher threshold for sick leave utilization due to illnesses not directly attributable to Sars-CoV-2 infection was possible because of the need to respond to the emergency caused by COVID-19 (47). Sickness absence due to neck and/or back disorders among primary HCWs in Qatar was similar in 2019 and 2020, but was significantly higher in the second wave than in the first wave of COVID-19 (48). On the other hand, we find more data in the literature on the prevalence of MSDs in HCWs during the COVID-19 pandemic. Musculoskeletal pain in the last 7 days was confirmed by 65% of respondents, with 54% reporting the most severe pain in the lower back (39). 73.9% of nurses reported the presence of MSDs symptoms in the last one-year period, with the most common complaint being lower back pain (42%) (40). During the COVID 19 pandemic, 63.9 of dental staff had symptoms of lower back pain over a 12-month period (41).

MSDs are a major cause of work-related morbidity and require the development of strategies to reduce the related absenteeism. Effective management of MSDs improves the health of the workforce and strengthens the resilience of the healthcare system, especially in the face of global health crises. Targeted measures based on the demographics of the healthcare workforce can optimize these outcomes. It would be useful to place a stronger focus on ergonomics and optimizing the work environment in times of high stress, such as during a pandemic. Attention should also be paid to potential presenteeism.

### 4.1 Limitations and perspectives

Several limitations of this study must be acknowledged. While the data allow for pre-pandemic and pandemic comparisons, they do not consider temporal variations due to other factors that might influence MSDs independently of the pandemic. The study relies on aggregated data that could mask individual differences or unique experiences. The study findings are based on Slovenian national data and may not be generalisable to HCWs in other countries with different healthcare systems or responses to the pandemic, although countermeasures to contain the spread of COVID-19 were likely similar in most western countries. The role of long-COVID-19 syndrome and SARS-CoV-2 vaccination were not considered. The number of previous SARS-CoV-2 infection was not clarified. Future studies could consider interventional approaches to identify effective strategies to reduce MSDs among HCWs during health crises, particularly in the area of ergonomics and optimizing the work environment.

## 5 Conclusion

This study examined the impact of the COVID-19 pandemic on the incidence and duration of musculoskeletal disorders (MSDs)-related sick leave (SL) among healthcare workers (HCWs). During unprecedented health crises such as the COVID-19 pandemic, different groups of HCWs show distinct SL patterns compared to the pre-pandemic period. Older HCWs are particularly vulnerable to shifts in SL due to MSDs. In this population, the average SL incidence decreased, but the duration of SL increased during the pandemic. This suggests that older HCWs on SL for MSDs generally had a more difficult disease course. Interestingly, the mere incidence of SL during a pandemic may not give a complete picture of the prevalence of non-infectious diseases such as MSDs. The underlying dynamics may be obscured, leading to underestimation or misunderstanding. For example, despite a consistent SL incidence across pandemic years, younger male HCWs have on average a shorter MSDs-related SL compared to their female counterparts. This discrepancy may indicate an insufficient recovery period for young male HCWs, potentially posing long-term health risks. With MSDs being one of the most common work-related diseases, it is crucial for healthcare managers to develop strategies and minimize MSDs-related absenteeism. This not only promotes a healthier workforce, but also a more resilient healthcare system, especially during global health emergencies. Tailored interventions targeting specific HCWs demographic groups could help achieve these goals.

## Data Availability

The raw data supporting the conclusions of this article will be made available by the authors, without undue reservation.

## References

[1] Jacquier-Bret J Gorce P. Prevalence of body area work-related musculoskeletal disorders among healthcare professionals: a systematic review. Int J Environ Res Public Health. (2023) 20:841. doi: 10.3390/ijerph20010841, PMID: 36613163 PMC9819551

[2] Yasobant S Rajkumar P. Work-related musculoskeletal disorders among health care professionals: a cross-sectional assessment of risk factors in a tertiary hospital, India. Indian J Occup Environ Med. (2014) 18:75. doi: 10.4103/0019-5278.146896 25568602 PMC4280781

[3] Andersen LL (2020) Musculoskeletal disorders in the healthcare sector. Discussion paper from European Agency for Safety & health at work. Available at: (https://osha.europa.eu/en/publications/musculoskeletal-disorders-healthcare-sector).

[4] World Health Organization (2023) Coronavirus disease (COVID-19) pandemic. Available at: https://www.who.int/europe/emergencies/situations/covid-19 (Accessed August 08, 2023).

[5] Spiteri G D’Agostini M Abedini M Ditano G Collatuzzo G Boffetta P. Protective role of SARS-CoV-2 anti-S IgG against breakthrough infections among European healthcare workers during pre and post-omicron surge—ORCHESTRA project. Infection. (2024) 52:1347–56. doi: 10.1007/s15010-024-02189-x, PMID: 38326526 PMC11289150

[6] Violán C Carrasco-Ribelles LA Collatuzzo G Ditano G Abedini M Janke C. Multimorbidity and serological response to SARS-CoV-2 nine months after 1st vaccine dose: European cohort of healthcare workers—Orchestra project. Vaccine. (2023) 11:1340. doi: 10.3390/vaccines11081340, PMID: 37631908 PMC10459685

[7] Ateş R Yakut H. Investigation of musculoskeletal disorders, physical activity level, sleep quality, and fatigue in health professionals with and without a history of COVID-19. Work. (2023) 74:1277–87. doi: 10.3233/WOR-220283, PMID: 36565091

[8] Ajab S Ádam B al M al N al M al A. Occupational health of frontline healthcare Workers in the United Arab Emirates during the COVID-19 pandemic: a snapshot of summer 2020. Int J Environ Res Public Health. (2021) 18:11410. doi: 10.3390/ijerph182111410, PMID: 34769927 PMC8583571

[9] Alzeyadi AA Elsiddig AI Khan MA Alkhaldi SA Alrumaym AH Alzaidi GA. Prevalence of musculoskeletal disorders among health care workers during covid-19 pandemic in the western region of Saudi Arabia. Med Sci. (2022) 26:ms104e2106. doi: 10.54905/disssi/v26i121/ms104e2106

[10] Arca M Dönmezdil S Durmaz ED. The effect of the COVID-19 pandemic on anxiety, depression, and musculoskeletal system complaints in healthcare workers. Work. (2021) 69:47–54. doi: 10.3233/WOR-205014, PMID: 34024799

[11] Sierpińska LE Ptasińska E. Evaluation of work conditions of nurses employed in a shift system in hospital wards during the COVID-19 pandemic. Work. (2023) 75:401–12. doi: 10.3233/WOR-220275, PMID: 36641728 PMC10357205

[12] Efe Is E Sahillioglu A Demirel S Kuran B Mustafa Ozdemir H. Effect of COVID-19 pandemic on physical activity habits, musculoskeletal pain, and mood of healthcare workers. Sisli Etfal Hastan Tip Bul. (2021) 55:462–8. doi: 10.14744/SEMB.2021.87523, PMID: 35317382 PMC8907692

[13] Mazal J Kelly N Johnson T Rose G Phelan D. Impact of COVID-19 on work-related musculoskeletal disorders for cardiac sonographers. J Am Soc Echocardiogr. (2021) 34:570. doi: 10.1016/j.echo.2021.01.007, PMID: 33422664 PMC7833236

[14] Adams R Branthwaite H Chockalingam N. Prevalence of musculoskeletal injury and pain of UK-based podiatrists and the impact of enforced altered working practices. J Foot Ankle Res. (2021) 14:53. doi: 10.1186/s13047-021-00491-7, PMID: 34470650 PMC8409074

[15] Eurostat. Statistical classification of economic activities in the European Community. Luxembourg: Eurostat (2008).

[16] Eurostat. Republic of Slovenia statistical office persons in employment by activities (NACE Rev. 2). Luxembourg: Eurostat (2008).

[17] Ishimaru T Yoshikawa T Okawara M Kido M Nakashima Y Nakayasu A. Presenteeism in front-line physicians involved in COVID-19-related clinical practice: a national survey of employed physician members of the Japan medical association. Environ Health Prev Med. (2023) 28:13–00194. doi: 10.1265/ehpm.22-00194, PMID: 36740269 PMC9922563

[18] White-Means SI Warren CL Osmani AR. The organizational impact of Presenteeism among key healthcare workers due to the COVID-19 pandemic. Rev Black Polit Econ. (2022) 49:20–40. doi: 10.1177/00346446211065175, PMID: 35291319 PMC8914299

[19] Challener DW Breeher LE Frain J Swift MD Tosh PK O’Horo J. Healthcare personnel absenteeism, presenteeism, and staffing challenges during epidemics. Infect Control Hosp Epidemiol. (2021) 42:388–91. doi: 10.1017/ice.2020.453, PMID: 33100247 PMC7684021

[20] Homrich PHP Dantas-Filho FF Martins LL Marcon ER. Presenteeism among health care workers: literature review. Rev Bras Med Trab. (2020) 18:97–102. doi: 10.5327/Z1679443520200478, PMID: 32783010 PMC7413686

[21] Nakua EK Otupiri E Dzomeku VM Owusu-Dabo E Agyei-Baffour P Yawson AE. Gender disparities of chronic musculoskeletal disorder burden in the elderly Ghanaian population: study on global ageing and adult health (SAGE WAVE 1). BMC Musculoskelet Disord. (2015) 16:204. doi: 10.1186/s12891-015-0666-3 26286129 PMC4541744

[22] Deslauriers S Déry J Proulx K Laliberté M Desmeules F Feldman DE. Effects of waiting for outpatient physiotherapy services in persons with musculoskeletal disorders: a systematic review. Disabil Rehabil. (2021) 43:611–20. doi: 10.1080/09638288.2019.1639222, PMID: 31304824

[23] Rhon DI Fraser JJ Sorensen J Greenlee TA Jain T Cook CE. Delayed rehabilitation Is associated with recurrence and higher medical care use after ankle sprain injuries in the United States military health system. J Orthop Sports Phys Ther. (2021) 51:619–27. doi: 10.2519/jospt.2021.10730, PMID: 34847698

[24] Ojha HA Wyrsta NJ Davenport TE Egan WE Gellhorn AC. Timing of physical therapy initiation for nonsurgical Management of Musculoskeletal Disorders and Effects on patient outcomes: a systematic review. J Orthop Sports Phys Ther. (2016) 46:56–70. doi: 10.2519/jospt.2016.6138, PMID: 26755406

[25] Alwabli Y Almatroudi MA Alharbi MA. Work-related musculoskeletal disorders among medical practitioners in the hospitals of Al’Qassim region, Saudi Arabia. Cureus. (2020) 12:e8382. doi: 10.7759/cureus.8382 32637265 PMC7331922

[26] Wijnhoven HAH de Vet HCW Picavet HSJ. Prevalence of musculoskeletal disorders Is systematically higher in women than in men. Clin J Pain. (2006) 22:717–24. doi: 10.1097/01.ajp.0000210912.95664.53 16988568

[27] Cimas M Ayala A Sanz B Agulló-Tomás MS Escobar A Forjaz MJ. Chronic musculoskeletal pain in European older adults: cross-national and gender differences. Eur J Pain. (2018) 22:333–45. doi: 10.1002/ejp.1123 29235193

[28] Overstreet DS Strath LJ Jordan M Jordan IA Hobson JM Owens MA. A brief overview: sex differences in prevalent chronic musculoskeletal conditions. Int J Environ Res Public Health. (2023) 20:4521. doi: 10.3390/ijerph20054521, PMID: 36901530 PMC10001545

[29] Srinivasan D Sinden KE Mathiassen SE Côté JN. Gender differences in fatigability and muscle activity responses to a short-cycle repetitive task. Eur J Appl Physiol. (2016) 116:2357–65. doi: 10.1007/s00421-016-3487-7, PMID: 27743025 PMC5118407

[30] Nordander C Ohlsson K Balogh I Hansson GÅ Axmon A Persson R. Gender differences in workers with identical repetitive industrial tasks: exposure and musculoskeletal disorders. Int Arch Occup Environ Health. (2008) 81:939–47. doi: 10.1007/s00420-007-0286-9, PMID: 18066574

[31] Bosma E Loef B van Oostrom SH Lifelines Corona Research Initiative Proper KI. The longitudinal association between working from home and musculoskeletal pain during the COVID-19 pandemic. Int Arch Occup Environ Health. (2023) 96:521–35. doi: 10.1007/s00420-022-01946-5, PMID: 36566457 PMC9790086

[32] Ministry of Health. Order on temporary measures to control the spread of the infectious disease SARS-CoV-2 (COVID-19). Ministry of Health of Republic of Slovenia. (2020).

[33] Terai H Tamai K Takahashi S Hori Y Iwamae M Ohyama S. The health-related quality of life of patients with musculoskeletal disorders after the COVID-19 pandemic. Int Orthop. (2022) 46:189–95. doi: 10.1007/s00264-021-05256-2 34735594 PMC8566965

[34] Dejaco C Alunno A Bijlsma JW Boonen A Combe B Finckh A. Influence of COVID-19 pandemic on decisions for the management of people with inflammatory rheumatic and musculoskeletal diseases: a survey among EULAR countries. Ann Rheum Dis. (2021) 80:518–26. doi: 10.1136/annrheumdis-2020-218697, PMID: 33158877

[35] Garrido-Cumbrera M Marzo-Ortega H Christen L Plazuelo-Ramos P Webb D Jacklin C. Assessment of impact of the COVID-19 pandemic from the perspective of patients with rheumatic and musculoskeletal diseases in Europe: results from the REUMAVID study (phase 1). RMD Open. (2021) 7:e001546. doi: 10.1136/rmdopen-2020-001546, PMID: 33827969 PMC8029094

[36] Liviero F Campisi M Mason P Pavanello S. Transient receptor potential Vanilloid subtype 1: potential role in infection, susceptibility, symptoms and treatment of COVID-19. Front Med. (2021) 8:753819. doi: 10.3389/fmed.2021.753819, PMID: 34805220 PMC8599155

[37] Liviero F Volpin A Furlan P Battistella M Broggio A Fabris L. The impact of SARS-CoV-2 on healthcare workers of a large University Hospital in the Veneto Region: risk of infection and clinical presentation in relation to different pandemic phases and some relevant determinants. Front Public Health. (2023) 11:1250911. doi: 10.3389/fpubh.2023.1250911 38098828 PMC10720910

[38] Campisi M Cannella L Bordin A Moretto A Scapellato ML Mason P. Revealing the hidden impacts: insights into biological aging and long-term effects in pauci- and asymptomatic COVID-19 healthcare workers. Int J Mol Sci. (2024) 25:8056. doi: 10.3390/ijms25158056, PMID: 39125624 PMC11311509

[39] Ciężarek P Frankowski G Micek A Zyznawska J Bonior J Wilk-Frańczuk M. Assessment of workload and pain in hospital workers using the standardized Nordic questionnaire-NMQ during the COVID-19 pandemic – a cross-sectional study. Pielegniar XXI Wieku. (2024) 23:124–9. doi: 10.2478/pielxxiw-2024-0023

[40] Chowdhury U Das T Mazumder S Gangopadhyay S. Work-related musculoskeletal disorders and mental health among nursing personnel in the context of COVID-19 pandemic in West Bengal, India. Int J Occup Saf Health. (2023) 13:234–44. doi: 10.3126/ijosh.v13i2.47775

[41] al-Hourani Z Almhdawi KA AlBakri IA Alibrahim A Obeidat D. The health and quality of life of dental workers in Jordan during COVID-19: a cross-sectional study. Work. (2024) 79:15–23. doi: 10.3233/WOR-220458, PMID: 38251081

[42] De Kok J VroonHhof P Snijders J Roullis G Clark M Peerebom K. European Agency for Safety and Health at Work: Work-Related Musculoskeletal Disorders: Prevalence, Costs and Demographics in the EU European Risk Observatory Report. (2020). Available at: https://osha.europa.eu/sites/default/files/Work_related_MSDs_prevalence_costs_and_demographics_in_EU_summary.pdf (Accessed March 04, 2024).

[43] Hämmig O. Work- and Stress-Related Musculoskeletal and Sleep Disorders among Health Professionals: A Cross-Sectional Study in a Hospital Setting in Switzerland. *BMC Musculoskelet*. *Disord*. (2020) 21:319. doi: 10.1186/s12891-020-03327-w 32438929 PMC7243303

[44] Nguyen LH Drew DA Graham MS Joshi AD Guo CG Ma W. Risk of COVID-19 among Front-Line Health-Care Workers and the General Community: A Prospective Cohort Study. Lancet Public Heal. (2020) 5:e475–83. doi: 10.1016/S2468-2667(20)30164-X PMC7491202 32745512

[45] Abdul Rahim H Fendt-Newlin M Al-Harahsheh S Campbell J. Our Duty of Care: A Global Call to Action to Protect the Mental Health of Health and Care Workers. Qatar: Doha (2022). Available at: https://www.who.int/publications/m/item/wish_report (Accessed March 07, 2024).

[46] Garbin AJÍ Nascimento CCMP Zacharias FCM Garbin CAS Moimaz SAS Saliba NA. Sickness Absenteeism of Primary Health Care Professionals before and during the COVID-19 Pandemic. Rev. Bras. Enferm. (2022) 75. doi: 10.1590/0034-7167-2022-0028 36043603

[47] Edge R van der Plaat DA Parsons V Coggon D van Tongeren M Muiry R. Changing Patterns of Sickness Absence among Healthcare Workers in England during the COVID-19 Pandemic. *J. Public Health (Bangkok)*. (2022) 44:e42–50. doi: 10.1093/pubmed/fdab341 PMC8499865 34514506

[48] Al-Nuaimi AA Abdeen S Abed Alah M AlHajri S Semaan S Al-Kuwari MG. Sickness Absenteeism among Primary Health Care Workers in Qatar before and during the COVID-19 Pandemic. *J. Occup. Med. Toxicol*. (2023) 18. doi: 10.1186/s12995-023-00369-3 36927778 PMC10018637

[49] Prijon T. Zdravstveni Absentizem Zaradi z Delom Povezanih Kostno-Mišičnih Obolenj in Duševnih Stresnih Motenj v Sloveniji. Primerjalna Analiza Začasne Nezmožnosti Za Delo v Letih 2015 in 2019 v Okviru Projekta “Promocija Aktivnosti Za Preprečevanje Kostno-Mišičnih. Ljubljana: (2020) (https://nijz.si/wp-content/uploads/2023/01/pkmo_analiza_bs_zaradi_z_delom_povezanih_kmo_in_dusevnih-stresnih_motenj.cleaned.pdf (Accessed January 28, 2024).

